# Lectures on Military Surgery

**Published:** 1861-08

**Authors:** E. Andrews

**Affiliations:** Professor of Surgery, Surgeon to the Mercy Hospital; to the Chicago Med. Dispensary, Etc.


### CHICAGO MEDICAL EXAMINER

N. S. DAVIS, M. D., Editor.---FRANK W. REILLY, M. D., Ass't Editor.

VOL. II.] AUGUST, 1861. [NO. 8.

### Original Contributions.

### ARTICLE I.

### LECTURES ON MILITARY SURGERY:

DELIVERED DURING THE SUMMER COURSE OF THE MEDICAL DEPARTMENT OF LIND UNIVERSITY.

By E. ANDREWS, A. M., M. D.,

PROF. OF SURGERY; SURGEON TO THE MERCY HOSPITAL; TO THE CHICAGO MED. DISPENSARY, ETC.

### LECTURE FOURTH.

### THE SURGEON ON THE FIELD OF BATTLE.

The previous lectures have, of necessity, been confined to the organization and machinery of the Medical Department of the army; but now we come to the more interesting topics of the uses of that organization, and the duties of the surgeon in operating that machinery.

Battles occur under all conceivable forms and circumstances, obliging the surgeons to vary their arrangements for the wounded according to the shifting scenes of the day. As a general rule, however, the following arrangements may be calculated upon in a field fight. The troops are arranged on a line from right to left, with space in the rear for free lateral communication to any part of the force. Somewhere in the rear of the line or lines, thus formed, a reserve force is stationed, ready to support any part of the army that may be too hardly pressed by the enemy.

The surgeon will locate his field hospital, if possible, in some spot out of the probable range of fire, and where it will not be likely to be in the way of movements of troops, especially of cavalry and artillery. Such spots, of course, will be in the rear of the line of battle, at any distance not too great for the ready succor of the wounded. If the formation of the ground permits, a place may be chosen behind the crest of a hill, where the elevation in front will be a safe protection from shot. A yellow flag is put at this place, or near it, as a guide for the wounded and their carriers, also as a notification to the enemy of the situation of the hospital, in order that they may avoid firing upon it. If there is a barn or house in the vicinity, it will be convenient to use it for a hospital, if not, a tent may be pitched, or even the shelter of trees resorted to. There should be a sufficient number of men detailed to the hospital, before the battle, to bring in the wounded as fast as they fall, so that on the one hand the line of battle is not weakened by men leaving their ranks to carry back the wounded, or on the other, the latter do not, by remaining too long unaided in the line, dispirit and alarm the men.

A portion of the surgeons remain at the temporary hospital to receive and attend to the wounded as they are brought in, and another party may in many instances follow close after the line of battle, to give such succor as may be necessary before the men are carried back. Each surgeon is attended by an orderly, who keeps close to him wherever he moves, and carries in a knapsack such instruments and medicines as are likely to be hastily called for. The contents of the knapsack are as follows:

CONTENTS OF HOSPITAL KNAPSACK.

| | |
|---|---|
| 1 quart Castor Oil. | 1 oz. Zinci Sulph., (Squibb's.) |
| 1 ℔. Cerati Simplicit. | ½ oz. Argenti Nitr., fused. |
| 4 oz. Chloroform, (Squibb's.) | 2 oz. Alum, (Squibb's.) |
| 4 oz. Ext. Ipecac. | 2 yds. Emp. Ichthyocollæ. |
| 4 doz. Pills, Cathartic, Comp. | 2 yds. Emp. Adhesivi. |
| 2 " " Mass. Hydr. grs. v. | ½ lb. Lint. |
| 2 " " Opii. | 2 pieces Sponge. |
| 4 " " " grj, Camph. grsij. | 4 doz. Bandages, assorted. |
| 4 " " Quinine Sulph. grs iij. | 2 yds. Flannel. |
| 1 oz. Potass, Iodide. | 1 Field Tourniquet. |

½ oz. Quinine Sulph.
4 oz. Spts. Ether, Comp. (Sqibb's.)
4 oz. Spts. Terebinth.
4 oz. Tinct. Opii.
4 oz. Tinct. Opii, Camph.
4 Binder's Boards, (4x18 inches,) doubled.
2 Lead Pencils.
1 quire Note Paper.
1 paper Pins and 1 p'ce Tape.

### AMBULANCES.

A large part of the wounded are only slightly injured, and are perfectly able to walk without assistance to the field hospital, but those who are at once disabled and fall in the line must either lie on the field till after the battle, or be carried off by the *Ambulance Corps.* The term "ambulance" is sometimes used to designate the whole organization and material of a flying hospital, and at others is restricted simply to the vehicles used for carrying the wounded.

The ambulance wagons are made either for two or four horses, and vary in their capacity. They will accommodate from two to eight patients. They are made with boxes of about the length of ten feet, set on springs, and covered with an oil-cloth to shelter the inmates from the sun and rain. There is a seat or two in front for the driver and for patients who are able to sit up. Behind the seats one or two tiers of berths are provided. These are covered with India rubber cloth that they may not be soiled with the blood, and are made so that they can be drawn out at the hinder extremity of the vehicle, thus the patient can be put in or removed without rising from his couch. In placing the wounded man in the ambulance, a berth is drawn out and placed upon the ground to receive the patient. It is then slid carefully into its place again, precisely like a drawer, with the head of the berth towards the rear end of the ambulance. Between the berths and the end of the vehicle there is a space of about eighteen inches, in which is placed a water cask.

"Ambulance carts" are constructed on similar principles, but with only two wheels. They are often of use in difficult routes, where heavier vehicles cannot proceed. The two-wheeled ambulance is considered best for patients who are very dangerously wounded. The number of these vehicles required in an army is one two wheeled ambulance for every company, and one four wheeled ambulance in addition, for every five companies.

### PANNIERS.

If the route of the command is over a region impassable to wheeled vehicles, the sick and wounded must be carried upon horses or mules. For this purpose, a long, secure saddle is constructed, to either side of which is attached a couch. One of these is made for placing the patient in a horizontal and the other in a sitting posture. In this way the parties are carried over mountain passes, or through forests where only bridle-paths exist.

### HORSE-LITTERS.

A more comfortable conveyance for the patient is the "Horse-Litter." This is composed of a canvas bed, attached by the sides to two poles, each sixteen feet long. There are head and foot pieces to keep the poles asunder. The poles are of ash, two and a half inches in diameter. The head and foot pieces have canvas "dashers" rising from them, stretched on strong wire, and serving for head and foot boards to the bed. The width of the canvas is twenty-seven inches, and the length five feet, ten inches. The litter thus constructed, is carried by placing a horse or mule between the poles at either end, and attaching the extremities to a suitable harness resting across the animal's back. The horse-litter is easier for the wounded than the panniers, and is better adapted for narrow forest paths, but it requires more beasts for the service.

### HAND-LITTERS OR STRETCHERS.

The United States hand-litters consist of a frame of ash with canvas stretched between. The side poles are eight feet ten inches in length, and an inch and a quarter in thickness. The cross pieces are twenty-nine inches in length, and placed one foot five inches from either end. The projecting poles serve as handles for the carriers. Hand-litters are often extemporized by cutting poles of suitable length and size, and fastening a blanket to them. Another simple litter is constructed by fastening a soldier's overcoat between two muskets.

The guns are passed in through the sleeves, and the front of of the coat is buttoned together so as to make a bed between the guns. If litters are extemporized for carrying men any great distance, cross pieces should always be lashed on to keep the poles apart, otherwise the litter will press against the hips of the carriers, and annoy them intolerably on the march.

### MANAGEMENT IN BATTLE.

The heavy ambulance wagons will generally remain either with the baggage train or at such a distance from the line of battle as not to become entangled in the movements of the troops, nor too much endangered by the fire. A sufficient number of men are detailed to carry hand-litters. As fast as men fall in the ranks these carriers place them upon the litters and carry them back to the field hospital, where they are attended by the surgeons detailed for that service. Some, however, are wounded in such a manner as to require instant attention on the spot. To provide for these, a suitable number of surgeons, with their orderlies, will keep near the line, ready to go wherever called, but in general taking care not to expose themselves to more fire than is required by their duties, as the loss of the surgeon on the field disheartens the men.

In sieges, the surgeon can advance into the trenches and dress the wounded on the ground before sending them to the rear.

If a fort is bombarded, the surgeon may find it requisite to place the hospital in a bomb-proof casemate, in order to protect the patients from shells. If a town is besieged, the hospital should be located, if possible, out of the line of the heaviest fire, and its situation pointed out to the enemy by a hospital flag.

Great care should be taken by the surgeon to provide for a full supply of water during the battle. Not only is this required for dressing and cleansing wounds, but also still more urgently for drink for the wounded. The loss of blood occasions a most agonizing thirst, which is frequently aggravated by the heat of the weather, and by the fact that the soldier may have fought for many hours already, without an opportunity to replenish his canteen. From these causes it happens that often the great cry of the wounded is for water —*water*. The men who carry the litters should keep their canteens constantly filled, and the field hospital itself should be fully provided for, either from springs, streams, or from casks previously brought to the spot and filled. The chief surgeon of the corps—called the Medical Director—assigns his subordinates to their several posts, and takes his own position at the field hospital, or "Ambulance Depot," as it is called. If the progress of the enemy endangers the safety of the Depot, the Quartermaster should send additional guards, or remove it to a safer position, according as the commanding officer may direct.

### INSTRUMENTS.

In Military Surgery some instrumental conveniences must be dispensed with for the sake of portability and simplicity. The following lists contain the instruments furnished by the government to each surgeon and assistant:

#### MISCELLANEOUS.

1 Sponge-holder, for the throat (Buck's.)
8 Cupping Glasses.
8 Tin Cups, for the same purpose.
6 Thumb Lancets (in cases.)
1 Spring Lancet.
1 Set of Pocket Instruments.
12 Whalebone Probangs.
4 Scarificators.
1 Set of Splints (major) Welch's.
1 Stomach Pump and case.
4 Enema Syringes, of which
 1 Davidson's,
 1 Hard Rubber, 6 oz.
8 Glass Penis Syringes.
8 India-rubber Penis Syringes.
1 Set of Teeth Extracting Instruments.
1 Tongue Depressor (on a hinge.)
8 Field Tourniquets.
2 Spiral Tourniquets (Petit's.)
6 Hernia Trusses.

#### AMPUTATING.

1 Capital Saw.
1 Metacarpal Saw.
1 Capital Amputating Knife.
1 Medium " "
1 Small " "
1 Large Catlin.
1 Small "
1 Scalpel.
1 Tenaculum.
1 Artery Needle.
1 Artery Forceps.
1 Bone Forceps.
1 Spiral Tourniquet.
12 Surgeon's Needles.
1 Mahogany Case, brass bound.
1 Gutta Percha Pouch.

TREPHINING.

| | |
|---|---|
| 2 Trephines. | 1 Elevator. |
| 1 Scalpel, with Raspitor. | 1 Brush. |
| 1 Hey's Saw. | 1 Mahogany Case, brass bound. |

EXSECTING.

| | |
|---|---|
| 1 Bone Forceps (Liston's.) | 1 Lenticular Knife. |
| 2 " " sharp, assorted. | 2 Spatulas, protecting. |
| 1 " " for sequestra. | 1 Trephine, small crown. |
| 1 Chain Saw. | 1 Ecraseur. |
| 1 Chisel. | 1 Mahogany Case, brass bound. |
| 1 Gouge. | 1 Gutta Percha Pouch. |

GENERAL OPERATING.

| | |
|---|---|
| 1 Metacarpal Saw. | 3 Scalpels. |
| 1 Trocar. | 1 Cataract Knife. |
| 1 Ball Forceps. | 1 " Needle. |
| 1 Gullet " | 1 Tenaculum. |
| 1 Artery " | 1 Double Hook. |
| 1 Dressing " | 6 Steel Bougies, silvered, double curve, Nos. 1 and 2, 3 and 4, 5 and 6, 7 and 8, 9 and 10, 11, 12. |
| 2 Scissors, straight and curved. | 6 Wax Bougies, Nos. 2, 4, 6, 8, 10. |
| 1 Artery Needle, with 4 points. | 3 Silver Catheters, Nos. 8, 6, 9. |
| 12 Surgeon's Needles. | 6 Gum-elastic Catheters, Nos. 1, 3, 5, 7, 9, 11. |
| 1 Tourniquet. | 2 Mahogany Cases, brass bound. |
| 1 Small Amputating Knife. | 1 Gutta Percha Pouch. |
| 1 " Catlin. | |
| 3 Bistouries. | |
| 1 Hernia Knife. | |

POCKET.

| | |
|---|---|
| 1 Large Scalpel. | 1 Tenaculum. |
| 1 Small " | 1 Tenotomy Knife. |
| 1 Artery Forceps. | 1 Abscess Lancet. |
| 1 Bull-dog Forceps. | 1 Exploring Needle. |
| 1 Curved " | 1 Exploring Trocar. |
| 1 Dressing " | 1 Seton Needle. |
| 1 Needle. | 1 Spatula. |
| 1 Sharp-pointed Bistoury. | 2 Probes. |
| 1 Probe-pointed " | 1 Director. |
| 1 Long Probe-pointed Bistoury. | 1 Double Canula. |
| 1 Straight Scissors. | 1 Comp'd Silver Catheter. |
| 1 Knee " | 6 Surgeon's Needles. |
| 1 Flat-curved Scissors. | 1 Artery Needle. |
| 1 Gum Lancet. | 1 Morocco Case. |

These remain in the hands of the medical officer so long as he continues in the service, but he is required to give them up on leaving.

Bandages should be made of the same material as in civil surgery, and should be rolled hard and put up in assorted packages of seven dozen each. Each package may be made up of the following sizes:

| | | | | | | | |
|---|---|---|---|---|---|---|---|
| 1 dozen | 1 inch | wide, | 1 yard | long. | | | |
| 2 | " | 2 | " | " | 3 | " | " |
| 2 | " | 2½ | " | " | 3 | " | " |
| 1 | " | 3 | " | " | 4 | " | " |
| ½ | " | 3½ | " | " | 5 | " | " |
| ½ | " | 4 | " | " | 6 | " | " |

Each roll should have its length and width marked upon it. The lint used in the service consists of scraped, ravelled and patent lint, cotton batting and wadding, and fine tow or beaten flax. The other furniture, dressings and medicines of the hospital are drawn according to certain established rules, and care is taken that every article be good and reliable.

### GUN-SHOT WOUNDS.

The projectiles which cause gun-shot wounds, present a great variety, and require a brief description.

*The Musket and Rifle Ball.*—These are either round or conical. The old spherical ball weighed about one ounce, and in preparing the cartridge three buck-shot were enclosed with it. The minie, or conical bullets of modern warfare, are intended for shooting in rifled barrels. They are elongated, coming to a blunt point in front, and having a funnel-shaped hollow at the base. Hence, the heaviest weight being in front, the bullet flies head on without upsetting, the lightened base acting on the air exactly as does the feather of an arrow. Another and still more important use of the hollow base, is that the expansion of the powder expands it, pressing the lead into the rifle grooves, and at once securing the rotation of the ball, and preventing the leakage of gas, called windage. From these various reasons the elongated balls fly with much greater accuracy and force than the spherical, and as we shall see hereafter, make more deadly and destructive wounds.

*Cannon Shot.*—These are either round or elongated as in the musket. They are also solid or hollow—the latter being called shells. The shells are intended to burst when they strike, and destroy life by the scattering of their fragments. Small shells are thrown by hand, and called hand-grenades. In artillery, canister and grape shot are also used. They consist of a number of small balls in one cartridge, which are all fired together, like the shot from a fowling piece. They are intended for close action. Solid shot often throw splinters of wood and fragments of stone from the objects which they strike, which thus become projectiles, producing distructive results.

### CHARACTERISTICS OF GUN-SHOT WOUNDS.

*Powder Marks.*—When a musket or other firearm is fired within a very short distance of the person, there is produced a blackened area of some inches in diameter, surrounding the bullet wound. This area is formed by unburned grains of powder and particles of carbon, which are projected like minute shot against the skin, and oftentimes imbed themselves within it. Thus lodged, the saltpetre and the sulphur are gradually dissolved and absorbed, while the carbon being insoluble, remains dispersed among the fibres of the dermis. Being too small and unirritating to cause suppuration, and thus procure their own release, they remain as permanent black spots on the skin, often greatly disfiguring the person. The proper remedy in this case, is to proceed patiently and thoroughly to extract each grain with some sharp instrument, as the point of a penknife. It has been recommended to apply corrosive sublimate to the skin, in sufficient strength to cause a superficial scab, which, when it falls off, will bring away the grains of powder entangled in it. I have not tried this, but I should fear the production of an ugly scar, unless great care were taken.

*Bullet Wounds.*—Where a bullet enters, it generally leaves a small, depressed, circular opening, of considerably less diameter than the projectile which made it. A small circular piece of skin is sometimes driven in before the ball. If the ball passes through the body, there is generally a difference in the appearance of the two orifices; for, while the place of entry shows a small, depressed and circular wound, the point of exit displays a larger opening, with more ragged and everted edges. The skin, at the latter place, seems often to open in a straight line, making a sort of button-hole wound. Often, several wounds may be made by the same shot, as, for instance, where it passes through both arms and the intervening chest. One of our soldiers at Cairo was accidentally shot in such a way that the same ball passed through both thighs and his head. This curious result, was owing to his being in a bent position at the time of receiving the discharge, —his head being down beside the thigh. The curious course of bullets in the human body, has long been a topic of remark. The old-fashioned round bullet, especially, when its force is nearly spent, is liable to be deflected from its direct line by every bone, tendon, or fascia, against which it impinges. Balls striking the chest, will sometimes glance from a rib and run half around the body, between the deep and superficial fascia, and make an exit opposite the point of entrance, presenting to the eye the appearance of having gone straight through the chest. The loose tisue of the interfascial space, affords so little resistance that it becomes the favorite track of balls, of which fact, great numbers of remarkable examples present themselves to military surgeons. One soldier was struck upon the larynx by a spent ball, which traversed completely around the neck, beneath the integument, and was found lying in the orifice where it entered. Another received a ball on the outer side of the right arm. Getting into the interfascial space, the projectile ran up the arm, around the bulge of the shoulder, obliquely down the back, and was cut out under the skin of the left thigh. Hence, it is obvious that the direction which a ball had, on entering the body, is no indication of its course. These remarks apply to the old round bullets, but the modern conical balls are much less eccentric in their route. As a general rule, they pass completely through the body, in a straight course, crushing bones, piercing fasciæ, and making very destructive wounds. The hæmorrhage in these, as in most other gun-shot wounds, is generally slight, but in a few instances, where a large vessel is cut, it is profuse and fatal. In striking the shafts of long bones, the bullet either glances off, or shatters the bone. In perforating the skull, or other flat bones, it makes a round opening, smaller on the side of entry and larger at the exit. The track of the ball is often more or less occupied with foreign bodies. Pieces of clothing, buttons, fragments of skin, and pieces of bone, are among the objects found. A thin layer of flesh, along the course of the wound, is usually pulpified and rendered gangrenous, hence, the wound rarely unites otherwise than by suppuration and granulation.

The wounds produced by cannon shot, are of course larger and more frightful than those of bullets. If they strike a limb, they tear it off, or, if they impinge upon any other part of the body, they make an equally terrific destruction. There is a peculiar class of injuries from cannon balls which has been called the wind-contusion. In these cases the skin is not broken, but the parts beneath are crushed, and in some instances, reduced to a complete pulp, even the bones being broken to fragments. The uninjured state of the skin rendered it difficult to conceive that there had been an actual contact of the shot, and hence sprung up the notion that the bruise was caused by the "wind of the ball." Recent experiments have shown that the wind of the ball is not capable of producing the slightest injury, and at the present time the wind contusion is believed to be due to the oblique stroke of a spent cannon ball, which having ricocheted along the ground, has acquired a rotary motion, and, in a manner, *rolls* across the person, crushing but not tearing.

Shells, grenades and rockets produce wounds by the fragments and the enclosed missiles thrown out in bursting. In general, they are comparable to musket shots, except that the fragments of shell being larger and more angular in their form, the laceration is more extensive.

*The shock* following a gun-shot wound is severe, being about equal, when large limbs are struck, to the depression following railroad accidents. The sensation produced by a bullet wound, is similar to that of receiving a smart blow with a cane, while the smart from smaller projectiles, such as buckshot, is compared by soldiers to that of the sting of a bee.

EXAMINATION OF THE WOUNDS.

In gun-shot, as in other wounds, the aspect of the patient gives much valuable information. The amount of fright and complaint is no indication of severe injury. Some soldiers will run to the rear, bellowing lustily, from the slightest scratch, while others remain silent under mortal wounds; but the general appearance of collapse in the countenance, indicates injury of important organs, while the absence of it shows that the bullet, though striking the chest, the abdomen, or the head, has passed by without touching any vital part.

In examining these cases, after noticing the amount of shock, attention should be turned to the hæmorrhage, if any exists, and lastly, to the course and situation of the ball, and the presence of foreign bodies. The course of the ball is often quite perplexing. The finger is a proper means of examining it at the orifice, but for deeper exploration a probe is required. Ordinary probes are often sufficient, but it will be necessary to have also a longer one, armed with a little ball at the extremity. With this, as full an investigation may be made as the nature of the case permits. At the same time, the presence of foreign bodies may be determined. If the track of the ball is too long or circuitous to be easily followed, the patient should not be tortured by excessive and wearisome probings. Frequently the ball will be found on the opposite side of the body, where it may be felt as a small hard tumor under the skin. The question of whether the lung is injured or not, is one of considerable importance in certain cases. This will be decided by ascertaining whether the air escapes more freely from the wound than it should from the mere influx and efflux from a wounded pleura, and also by noticing whether the patient expectorates any blood. Notice should be taken also, in cases of wounded lungs, whether the bullet passed quite through, or lodged in the substance of the organ. More patients survive who are shot completely through the chest, than of those in whom balls stop in the lung. Many patients recover from wounds penetrating the chest, while very few survive the passage of bullets through the abdomen. Gunshot wounds of the brain are almost uniformly fatal, although there are a few astounding recoveries on record.

Having thoroughly examined the patient, the surgeon will proceed to the treatment, which will be the subject of the next lecture.

